# Comparative efficacy of naltrexone-accelerated versus sublingual buprenorphine in opioid withdrawal management and initiating injectable naltrexone treatment: study protocol for a randomized controlled trial

**DOI:** 10.3389/fpsyt.2026.1933443

**Published:** 2026-10-07

**Authors:** Saeede Jafari, Afarin Rahimi-Movaghar, Alireza Noroozi, Pooneh Malekifar, Akbar Fotouhi

**Affiliations:** 1 Department of Epidemiology and Biostatistics, School of Public Health, Tehran University of Medical Sciences, Tehran, Iran; 2 Iranian National Center for Addiction Studies (INCAS), Tehran University of Medical Sciences, Tehran, Iran

**Keywords:** heroin, naltrexone depot formulations, opioid dependence, opioid detoxification, opium, parallel-group trial

## Abstract

**Background:**

Extended-release injectable naltrexone (XR-NTX), a long-acting opioid antagonist, is an effective relapse prevention strategy in the treatment of opioid use disorder (OUD). However, standard XR-NTX initiation requires completion of opioid withdrawal management and a 1–2 week opioid-free period. This interval is associated with heightened vulnerability to lapse and relapse, and many patients fail to initiate XR-NTX treatment successfully. Therefore, alternative strategies that enable rapid XR-NTX initiation are needed. This study aimed to compare the effectiveness and safety of naltrexone-accelerated (NA) versus sublingual buprenorphine (SB) withdrawal management in facilitating XR-NTX initiation among patients with OUD.

**Methods:**

This single-center, open-label, randomized controlled trial uses a parallel-group design. Eligible patients aged 18–60 years with moderate to severe OUD who are actively using opioids and are not receiving regular maintenance treatment with methadone or buprenorphine are randomly assigned to one of two groups: (1) NA withdrawal management, comprising one day of adjunctive non-opioid medications, one day of buprenorphine administration, one day of washout, and four days of gradual oral naltrexone titration (1, 3, 12, and 25 mg/day); or (2) SB withdrawal management, comprising one day of adjunctive non-opioid medications, six days of buprenorphine tapering (up to 8 mg on Day 2, 6 mg on Day 3, 4 mg on Days 4–5, 2 mg on Day 6, and 1 mg on Day 7), and a seven-day washout period. Adjunctive non-opioid medications in both groups comprised clonidine, clonazepam, gabapentin, and trazodone. The primary outcomes are the proportions of participants who (1) achieve a negative urine drug test for the assessed opioids and (2) receive XR-NTX at the end of the withdrawal management period. Secondary outcomes include severity of withdrawal symptoms, opioid craving intensity, and adverse effects. All analyses are conducted according to the intention-to-treat principle.

**Discussion:**

The findings of this trial may provide clinically relevant evidence to support rapid initiation of XR-NTX treatment in patients with OUD and inform clinical practice.

**Clinical trial registration:**

https://en.irct.ir/trial/81525, Iranian Registry of Clinical Trials IRCT20250123064499N1.

## 1 Introduction

Opioid use disorder (OUD) represents a major public health challenge worldwide. According to the most recent global estimates, in 2021, OUD accounted for the highest incidence and mortality among all substance use disorders (1), with approximately 1.94 million new cases (age-standardized incidence: 24.54 per 100,000) and 99.55 thousand deaths (age-standardized mortality rate: 1.19 per 100,000) (2).

According to the World Health Organization (WHO) guidelines, pharmacotherapy is the cornerstone of clinical management for many patients with OUD, primarily involving the use of opioid agonist maintenance treatment (OAMT) with medications such as methadone and buprenorphine (3). In Iran, OAMT has become the dominant approach for treating OUD over the past few decades and is widely provided through addiction treatment centers. Nevertheless, due to various clinical or personal factors, some patients may prefer antagonist-based treatments, such as naltrexone. Oral naltrexone, a competitive μ-opioid receptor antagonist, was approved in 1984 for the blockade of the effects of exogenously administered opioids (4). Despite its potential advantages (e.g., no abuse liability, no special regulatory requirements), oral naltrexone is not widely used to treat OUD because of high rates of medication nonadherence (5). Subsequently, an extended-release, monthly injectable formulation of naltrexone was developed and was approved for maintenance treatment of OUD in 2010 (6). Randomized clinical trials demonstrates the safety and efficacy of extended-release injectable naltrexone (XR-NTX) both versus placebo and compared with standard OUD treatments (7, 8). Nevertheless, initiating naltrexone treatment presents challenges related to opioid withdrawal management, particularly in outpatient settings (9–11). A safe and effective withdrawal management strategy is a critical step in abstinence-based approaches and can serve as the foundation for subsequent relapse prevention strategies.

Opioid withdrawal management is typically performed using opioid agonists such as buprenorphine, followed by a gradual taper over 7 to 21 days. However, to initiate naltrexone treatment, patients must abstain from opioids for 1–2 week to avoid precipitated withdrawal. This period may increase the risk of treatment noncompletion, relapse, and failure to transition to XR-NTX, particularly in outpatient settings, due to protracted withdrawal symptoms and cravings (4, 12, 13). In contrast, accelerated withdrawal management approaches, which are based on the combined use of an opioid agonist and antagonist, eliminate the washout period and enable rapid transition to XR-NTX. These methods are usually completed within approximately 7 days, and the induced withdrawal symptoms are commonly managed with adjunctive non-opioid medications such as clonidine, clonazepam, and trazodone (14–17).

Despite the limited and sometimes conflicting international evidence comparing the safety and efficacy of conventional and accelerated withdrawal management strategies (14–16), to our knowledge, no study on this topic has been conducted in Iran or other developing countries. Since all of the aforementioned studies have been carried out in developed countries, conducting such research in diverse communities with different patterns of OUD may help generate evidence from various regions of the world, enhance the external validity of the findings, and provide valuable insights for clinicians and addiction treatment specialists. Therefore, this study is designed to compare the effectiveness and safety of two withdrawal management strategies—naltrexone-accelerated (NA) withdrawal management versus sublingual buprenorphine (SB) withdrawal management—in facilitating successful XR-NTX initiation among patients with OUD. We hypothesize that NA withdrawal management will lead to higher rates of negative urine drug tests for the assessed opioids and successful XR-NTX initiation compared with SB management.

## 2 Methods

### 2.1 Study design and setting

This is a single-center, randomized, unblinded, two-arm parallel group clinical trial, which has commenced in April 2025 at the outpatient clinic of the Iranian National Center for Addiction Studies (INCAS), affiliated with Tehran University of Medical Sciences (TUMS), in Tehran, Iran.

### 2.2 Participants

Participants in this study are individuals diagnosed with moderate to severe OUD according to the Diagnostic and Statistical Manual of Mental Disorders, Fifth Edition (DSM-5), aged 18–60 years, and residing in Tehran. Participants must have evidence of recent opioid use based on a rapid urine drug test that detects morphine, tramadol, methadone, and buprenorphine. In Iran, the most commonly used opioids among individuals with OUD include opium, condensed extract of smoked opium ash (Shireh), and heroin, all of which can be detected by testing for morphine in urine. Tramadol, methadone, and buprenorphine are other frequently used opioids. Therefore, the chosen rapid urine drug test panel covers the opioids typically encountered in our clinical setting. Misuse of fentanyl and oxycodone is uncommon in the country. Participants must also be able to provide written informed consent.

Furthermore, participants must not currently be receiving regular OAMT with methadone or buprenorphine as part of an ongoing treatment plan from which they are benefiting, because participation in the study could disrupt their established clinical care and treatment plan. Individuals who are receiving OAMT but intend to discontinue it and undergo withdrawal management for transition to naltrexone treatment, as well as those who use methadone or buprenorphine for non-medical purposes, are eligible.

Additional inclusion criteria include the absence of current prescribed opioid medications (e.g., oxycodone) for chronic pain; the absence of severe or acute medical or psychiatric disorders; the absence of physiological dependence on alcohol or sedative/hypnotic medications; and the absence of a history of opioid overdose within the past 3 years. Furthermore, participants must have no history of allergy or hypersensitivity to naltrexone or XR-NTX components, including polylactic-co-glycolic acid, carboxymethyl cellulose, or other diluents. Finally, among the participants who meet the inclusion criteria, those with impaired liver function (ALT and AST > 3 times the upper limit of normal), coagulation disorders (platelet count < 50,000/µl and/or abnormal PT or PTT values), pregnancy, or breastfeeding are excluded from the study.

A study physician assessed eligibility using a researcher- designed checklist containing all predefined inclusion and exclusion criteria. The physician reviewed each criterion individually and documented whether it was met, using relevant clinical and paraclinical assessments when required. These assessments included DSM-5 diagnostic criteria, physical examination (e.g., signs of opioid intoxication or withdrawal and injection-site marks), and laboratory testing (urine, blood, and pregnancy testing), as applicable to the specific eligibility criterion.

### 2.3 Recruitment

A multi-modal recruitment strategy is implemented to inform potentially eligible candidates about the study. This includes publishing advertisements on the official INCAS website and social media channels, displaying informational banners within the clinic, distributing electronic announcements through specialized channels targeting addiction therapists in Tehran province, and displaying posters at healthcare facilities affiliated with TUMS and in public thoroughfares.

Potential participants are evaluated by a physician based on the study’s eligibility criteria. Subsequently, the physician provides comprehensive explanations concerning the participant’s diagnosis, the clinical consequences of opioid use, the nature of OUD, and the advantages and disadvantages of different therapeutic modalities. Study objectives and the nature of randomization in assigning participants to treatment groups are also explained, and adequate time is provided for participants to ask questions and make informed decisions. If an individual declines to participate, the reasons for non-consent, along with their baseline characteristics, are documented, and the individual is excluded from the study.

### 2.4 Randomization, allocation concealment, and blinding

On Day 1, after screening and obtaining informed consent, eligible participants are randomized in a 1:1 ratio to either NA (intervention group) or SB withdrawal management (comparison group). Balanced block randomization is performed using stratification by opioid-use category (heroin vs. non-heroin), determined by self-reported use and the results of urine drug tests for the assessed opioids. The heroin stratum comprises participants whose primary opioid is heroin, whereas the non-heroin stratum includes those using other opioids. Within each stratum, a fixed block size of four is used (**Figure 1**). The randomization sequence is generated by the randomization officer (PMF), who has no other involvement in study implementation, using Stata/SE 14.2 (StataCorp LLC, Texas, USA).

**Figure 1:**
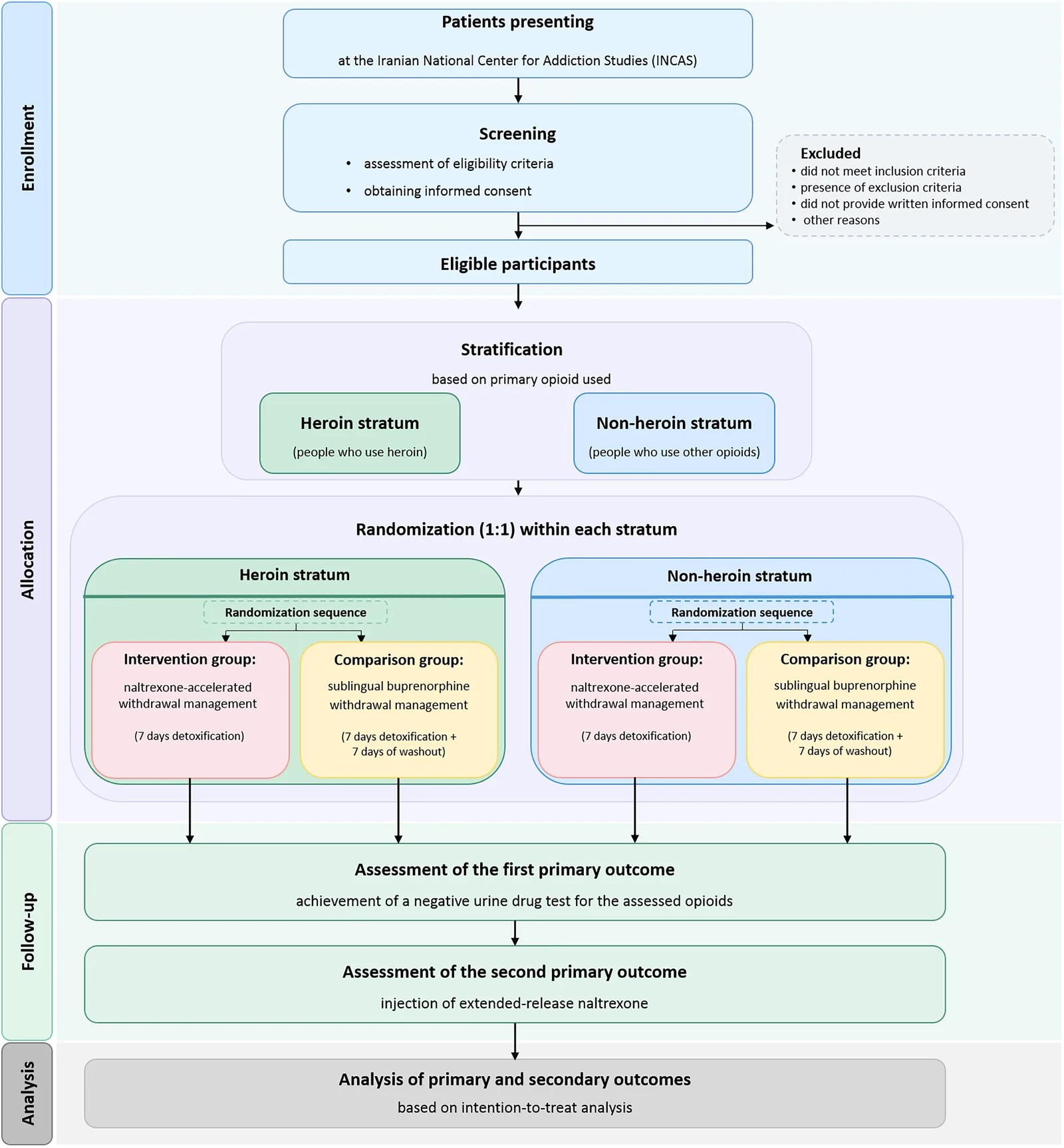
Study flow diagram.

To ensure allocation concealment and prevent potential manipulation of the randomization process, the randomization sequence is held exclusively by the PMF. The operational procedure is as follows: as eligible participants enter the study, the assessor assigns an entry number and then contacts the PMF, providing the entry number, full name, and opioid-use category. The PMF then communicates the assigned intervention group and its corresponding allocation code to the assessor for documentation in the case report form (CRF). Subsequently, the assessor informs the physician and the participant of the assigned intervention. Notably, this study is unblinded; all involved individuals are aware of the intervention received.

For audit trail and oversight, randomization process details—including participant entry number, allocation code, and intervention group—are separately documented by the assessor in dedicated forms. These forms are archived separately from the main CRF and made accessible to the study monitor for verifying allocation procedures against the randomization sequence as needed.

### 2.5 Primary outcomes

The primary outcomes are the proportions of participants who (1) achieve a negative urine drug test for the assessed opioids, and (2) receive XR-NTX at the end of the withdrawal management period. Both outcomes are recorded as dichotomous variables. In this study, the occurrence of any of the following events between randomization and administration of XR-NTX is defined as dropout: withdrawal of informed consent, discontinuation of treatment, or loss to follow-up.

### 2.6 Secondary outcomes

The secondary outcomes include severity of withdrawal symptoms, as measured by the Clinical Opiate Withdrawal Scale (COWS) and the Subjective Opiate Withdrawal Scale (SOWS), opioid craving intensity, treatment retention (defined as the percentage of participants attending scheduled visits and receiving and adhering to assigned medication), opioid use (quantified via self-report) during the withdrawal management period, safety (including the incidence of any adverse events associated with the withdrawal management strategy, exclusive of withdrawal symptoms as measured by COWS and SOWS), and patient-reported treatment satisfaction.

### 2.7 Sample size

Sample size calculation is based on the study by Shulman et al., considering the primary outcome of XR-NTX injection. This study reported an injection rate of approximately 63% in the NA group compared to about 35% in the SB group. Accordingly, to detect this difference with 80% statistical power and a Type I error of 0.05, a total sample size of 98 participants is estimated. With a 1:1 allocation ratio, each group comprises 49 participants.

### 2.8 Intervention

#### 2.8.1 General withdrawal management procedures

The withdrawal management process for the naltrexone group involves a 7-day period, while for the buprenorphine group, it includes a 14-day period, consisting of 7 days of pharmacotherapy and a 7-day washout period. Following the completion of these periods, patients in both groups receive XR-NTX (Exopio 380 mg, Nano Daru Co., Tehran, Iran) on the eighth and fifteenth days, respectively (**Table 1**). The commencement of the withdrawal management protocol for all participants is fixed on Saturday (the first working day of the week in Iran), and the intervention continues throughout the week. Participants attend the center daily in the morning during the intervention period, and treatment procedures are implemented according to the predetermined schedule from 9 AM to 3 PM. If the treatment period coincides with the weekend or official holidays, to ensure continuity of care, medications will be provided to patients as take-home doses after prior instruction on their usage, and clinical monitoring during these days will be conducted via telephone visits. This timing is designed to guarantee uniform protocol implementation, continuous monitoring of withdrawal symptoms, and timely management of potential adverse effects.

**Table 1:** Opioid withdrawal management medication schedule.

Day	NA withdrawal management	SB withdrawal management
1	Adjunctive non-Opioid Medications^*^	
2	Sublingual Buprenorphine up to 8 mg (2mg + 2mg + 2mg + 2mg)	
3	Washout	Sublingual Buprenorphine up to 6 mg
4	Oral Naltrexone 1 mg (0.5 mg + 0.5 mg)	Sublingual Buprenorphine up to 4 mg
5	Oral Naltrexone 3 mg (1.5 mg + 1.5 mg)	Sublingual Buprenorphine up to 4 mg
6	Oral Naltrexone 12 mg (6 mg + 6 mg)	Sublingual Buprenorphine 2 mg
7	Oral Naltrexone 25 mg (12.5 mg + 12.5 mg)	Sublingual Buprenorphine 1 mg
8	Extended-release injectable naltrexone 380 mg	Washout
15		Extended-release injectable naltrexone 380 mg

NA, naltrexone-accelerated; SB, sublingual buprenorphine.

*Adjunctive non-opioid medications: clonidine 0.1 mg every 8 h (max 0.6 mg/day), clonazepam 0.5 mg every 8 h (max 2 mg/day), gabapentin 300 mg every 8 h, and trazodone 50–100 mg at bedtime in case of insomnia. In the NA group, they are continued from Day 3 through Day 8. In the SB group, they may be prescribed during the washout period (Days 8–14) at the physician’s discretion.

#### 2.8.2 Initial withdrawal management (days 1–2)

In both study arms, the first and second days are identical. On the first day, adjunctive non-opioid medications are prescribed to prepare patients for the management of opioid withdrawal. Patients are instructed to abstain from any opioid use or opioid-based medications for 12 to 24 hours before the next day’s visit. With the onset of withdrawal symptoms, patients are advised to take the adjunctive non-opioid medications, including clonidine 0.1 mg every 8 hours (titrated up to a maximum of 0.6 mg per day), clonazepam 0.5 mg every 8 hours (titrated up to a maximum of 2 mg per day), gabapentin 300 mg every 8 hours, and trazodone 50–100 mg at bedtime in case of insomnia.

On the second day, following the cessation of opioid use from the previous day, the severity of withdrawal symptoms is assessed using the COWS. If the COWS score is ≥ 6, treatment is initiated for participants in both groups with an initial 2 mg of sublingual buprenorphine, followed—if symptoms persist—by additional 2-mg doses every 1 to 2 hours, up to a maximum of 8 mg. If the COWS score is < 6, the protocol involves waiting for the emergence of withdrawal symptoms when feasible; otherwise, initiation of treatment is postponed to the following day.

#### 2.8.3 NA withdrawal management

On Day 3, participants in the NA group begin the washout period, during this day, adjunctive non-opioid medications are prescribed according to the Day 1 regimen and continued through Day 8.

Oral naltrexone induction commences on Day 4 at 1 mg (following ondansetron treatment, 4–8 mg), with doses escalating to 3 mg, 12 mg, and 25 mg by Day 7. Oral naltrexone is prepared and administered as a solution. For this purpose, a 25 mg naltrexone capsule is dissolved daily in 250 ml of water to achieve a concentration of 0.1 mg/ml. Accordingly, a 1-mg dose is prepared by withdrawing 10 ml of the solution, a 3-mg dose by withdrawing 30 ml, a 12-mg dose by withdrawing 120 ml, and a 25-mg dose by withdrawing the entire 250 ml of the solution. The daily dose is divided into two parts and administered one hour apart. The decision to administer each portion of the solution is based on the assessment of withdrawal symptom severity using COWS. Before initiating the naltrexone solution, adjunctive non-opioid medications, including clonidine (0.1 mg) and clonazepam (0.5 mg), are started. After 1 to 3 hours, the COWS is assessed. If COWS ≤ 5, the first portion of the oral naltrexone solution is administered. One hour later, the COWS is reassessed, and if the score does not increase by more than 3 units, the second portion is administered. If symptoms intensify or the COWS score increases by more than 3 units, the second dose is withheld, symptoms are managed with adjunctive non-opioid medications, and the day’s protocol is postponed to the following day.

#### 2.8.4 SB withdrawal management

From Day 3 to Day 7, patients receive tapering doses of SB. The maximum dose on Day 3 is 6 mg, decreasing to 4 mg on Days 4 and 5, and further reducing to 2 mg and 1 mg on Days 6 and 7, respectively. Upon patient presentation to the clinic, a 2 mg SB tablet is administered (except on Day 7, when the total daily dose is 1 mg). If withdrawal symptoms persist, subsequent doses may be given at 1- to 2-hour intervals until symptoms resolve. Should symptoms resolve while a portion of the daily dose remains, the remainder is provided to the patient for home use, with instructions to take it as needed for symptom recurrence.

From Day 8 to Day 14, a washout period is implemented for this group. Patients are strongly advised to abstain from opioid use or opioid-based medications. If clinically indicated, adjunctive non-opioid medications may be prescribed at the physician’s discretion according to the Day 1 regimen.

#### 2.8.5 XR-NTX administration

XR-NTX is administered in both study groups based on an identical protocol; however, it is injected on Day 8 in the NA group and on Day 15 in the SB group. Prior to injection, completion of the withdrawal management period, absence of self-reported regular opioid use for at least 24 hours in the NA group and 7 days in the SB group, and a negative urine drug test for the assessed opioids are mandatory. Subsequently, a clinical assessment of withdrawal symptoms using the COWS is performed. If the patient’s COWS score is 4 or below, the antagonist challenge test is administered. If the challenge test result is negative, the XR-NTX is injected. Should any of the above conditions remain unmet, the patient’s symptoms will be managed with adjunctive non-opioid medications, and the attempt to administer the naltrexone injection will be repeated the following day (**Table 1**). All clinical, paraclinical, and medication-related services required for withdrawal management are provided free of charge to participants in this study. Under usual treatment conditions, out-of-pocket costs for withdrawal management at this center are minimal because it is a public facility and has agreements with most insurance plans. However, insurance does not cover XR-NTX under usual treatment conditions, and patients generally bear the full cost.

Patients who have completed the withdrawal management and received the XR-NTX are provided with adjuvant non-opioid medications for one week to alleviate residual withdrawal symptoms. Furthermore, patients are offered the opportunity to continue their routine treatment with XR-naltrexone for an additional 6 months at the study site.

#### 2.8.6 Naltrexone challenge test procedure

A diluted naltrexone solution (0.1 mg/ml), prepared by dissolving a 25 mg naltrexone capsule in 250 ml of water, is administered in four escalating doses: 1 mg (10 ml), 4 mg (40 ml), and two subsequent 10 mg doses (100 ml each). Following each dose, the patient is monitored for 30 minutes. If the patient tolerates the naltrexone well (meaning no withdrawal symptoms or an increase of less than 2 points on the COWS), the next dose is prescribed. Otherwise, the test is discontinued, and after investigating the cause of intolerance, the test is repeated 1–2 days later. A maximum of three test repetitions is permitted, and if the test is positive for the third time, the patient will be withdrawn from the study.

#### 2.8.7 Additional interventions

During the withdrawal management period, patients are educated regarding the risk of orthostatic hypotension, drowsiness, and decreased level of consciousness induced by clonidine. Blood pressure (BP) and heart rate (HR) are monitored daily, and continuation of clonidine administration is permitted only if BP is maintained at a minimum of 60/90 mmHg and HR is ≥55 bpm.

In this study, each participant receives two individual counseling sessions: one on the first day and one on the final day. Sessions are conducted by a trained psychologist according to a standard protocol using a dedicated checklist. Each session lasts 45 to 60 minutes and covers topics such as motivational interviewing and cognitive-behavioral therapy–based relapse prevention. If clinically indicated, the patient is referred to a psychiatrist for further specialized evaluation. The fidelity of the sessions is monitored through random and direct supervision by internal audit to ensure consistent delivery across all participants in accordance with the protocol.

Patients are advised by the physician to refrain from driving or operating hazardous machinery until their functional status is confirmed to be unimpaired. Furthermore, the risks of concomitant use of buprenorphine with other sedatives and alcohol are explained. Given the reduced tolerance following substance cessation, overdose prevention training is provided to all patients, particularly to those who prematurely discontinue treatment.

### 2.9 Follow-up during withdrawal management

During the withdrawal management phase, participants are actively followed up by the assessor. In the event of a missed appointment, patients are contacted via telephone. If a session is missed without concomitant opioid use, the treatment protocol is resumed upon the patient’s return. However, if a one-day absence occurs alongside opioid use, the detoxification program from the preceding day is repeated. For absences lasting two days or more accompanied by continuous opioid use, the detoxification schedule is restarted from the beginning. Should this pattern recur during a subsequent attempt, the participant is withdrawn from the study and referred for further clinical assessment to determine the need for OAMT.

### 2.10 Research team training

Therapists participating in this study have extensive experience in treating OUD. Nevertheless, all treatment personnel, including psychiatrists, physicians, psychologists, and laboratory technicians, as well as executive personnel, including assessors and an auditor, undergo supplementary training to ensure the correct implementation of study procedures and patient safety. The training includes theoretical and practical sessions. The content of the theoretical sessions covers familiarity with the study design and its objectives, methods for managing NA and SB withdrawal, assessment of withdrawal symptoms, management of side effects, data recording in the CRF, and precise audit implementation. Practical sessions are also assessed and confirmed through role-playing sessions.

### 2.11 Assessments

In this study, a comprehensive set of clinical and non-clinical variables is collected using a CRF that consists of a researcher-designed checklist and validated standardized questionnaires.

The researcher-designed checklist comprises several sections. The first section is dedicated to clinical assessment, including the recording of physical and psychiatric history, physical examination, vital signs (systolic and diastolic BP, HR, and body temperature), and anthropometric indicators (height and weight). Except for vital signs, which are assessed at all visits, all other assessments are performed by a physician at baseline. The diagnosis of moderate to severe OUD is also made by the physician using DSM 5 criteria.

The second section pertains to paraclinical tests, including a pregnancy test (for female participants), blood tests (CBC, ALT, AST, PT, PTT, and HIV), and a rapid urine drug-testing panel for opioids, as well as other substances (methamphetamine, benzodiazepines, and tetrahydrocannabinol). Pregnancy and blood tests are conducted at baseline, and the rapid urine drug test is performed by laboratory technicians at baseline and prior to the injection of XR-NTX.

The third section focuses on demographic characteristics, encompassing age, gender, marital status, education level, and employment status. The fourth section pertains to substance use history, encompassing the types of substances used, age of onset, injection history, and history of substance use disorder treatment. The third and fourth sections are gathered by the assessor using a face-to-face interview at baseline.

The fifth section includes treatment program information, covering data on visits, medication use, and adverse events, recorded by the assessor through observation during all treatment sessions.

Regarding standardized questionnaires, one of them is the Severity of Dependence Scale (SDS), which is used to measure opioid dependence severity. This tool includes 5 questions, each with 4 options, and each question scores between 0 and 3. The sum of the scores of these questions determines the final score of the index, which ranges from 0 to 15. A higher score indicates greater severity of dependence. Its validity and reliability have been demonstrated in Persian (18). SDS is administered by the assessor through a face-to-face interview at baseline.

The Numerical Rating Scale (NRS) is used to assess the severity of opioid craving at two time points: current craving at the time of the interview and the most severe craving experienced during the previous 24 hours. This scale ranges from 0 to 10, where 0 indicates no craving, and 10 indicates uncontrollable craving (19). Patients are instructed to rate their craving on this scale at each treatment session.

The COWS includes 11 clinical items such as resting HR, sweating, restlessness, pupil size, joint or bone pain, runny nose or tearing, gastrointestinal upset, shivering, yawning, anxiety or irritability, and piloerection. These items are scored from 0 to 4 or 0 to 5. The sum of the scores of these items determines the total score of this scale, which ranges from 0 to 48 (20). Its validity and reliability have been demonstrated in Persian (21). Prior to the administration of the primary medication, COWS is directly evaluated by the assessor at all treatment sessions.

The SOWS includes 16 subjective items such as anxiety, yawning, sweating, tearing, runny nose, piloerection, chills, hot flashes, feeling cold, musculoskeletal pain, restlessness, nausea, vomiting, muscle spasms, abdominal cramps, and drug craving. These items are scored based on a 5-point Likert scale (Not at all, Mild, Moderate, Severe, and Very Severe) from 0 for “Not at all” to 4 for “Very Severe.” The sum of these item scores determines the total score for this scale, which ranges from 0 to 64 (22). Prior to the administration of the primary medication, SOWS is completed by the patient through self-report at all treatment sessions.

The World Health Organization Quality of Life questionnaire (WHOQOL-BREF) includes 26 questions across four domains: physical health, psychological health, social relationships, and environmental health. Each domain contains 7, 6, 3, and 8 questions, respectively. The first two questions do not belong to any specific domain and assess overall health status and quality of life. The score for each domain is calculated separately, ranging from 4 to 20, representing the worst to best condition for the respective domain. Finally, these scores can be converted to a score ranging from 0 to 100 (23). The validity and reliability of the Persian version have been previously established (24). The WHOQOL-BREF is completed by the patient at baseline to characterize participants’ baseline quality of life and to provide a potential baseline covariate for consideration in the statistical analysis, if appropriate. Given the relatively short duration of the study, meaningful changes in quality of life might not be adequately captured within this timeframe. Therefore, in our study, the WHOQOL-BREF is not used to assess longitudinal changes in quality of life.

The Beck Depression Inventory (BDI) is used to assess the severity of depressive symptoms. This instrument consists of 21 items, each rated on a 4-point Likert scale from 0 to 3. The total score ranges from 0 to 63, with higher scores indicating more severe depression. Based on the total score, depression is categorized as minimal (0–13), mild (14–19), moderate (20–28), and severe (29–63) (25). The validity and reliability of the Persian version of the BDI have been demonstrated (26). The BDI is completed by the patient at baseline to characterize participants’ baseline depressive symptom severity and to provide a potential baseline covariate for consideration in the statistical analysis, if appropriate. Given the relatively short duration of the study, meaningful changes in depressive symptoms might not be adequately captured within this timeframe. Therefore, in our study, the BDI is not used to assess longitudinal changes in depressive symptoms.

The Client Satisfaction Questionnaire (CSQ) is used to assess patients’ satisfaction with the provided services. This instrument includes 8 items, each rated on a 4-point Likert scale from 1 to 4. The total score ranges from 8 to 32, indicating the lowest to the highest degree of satisfaction, respectively (27). The validity and reliability of its Persian version have been demonstrated (28). The CSQ is completed by the patient on day 8 in the NA group and on day 15 in the SB group.

The timeline for each measure, along with the baseline assessments, is presented in **Table 2**.

**Table 2:** The timeline for measures in the intervention (NA) and the comparison (SB) groups.

Measurement (instrument)	Baseline (or Day 1)	Day 2	Day 3	Day 4	Day 5	Day 6	Day 7	Day 8	Day 15
Physician visit and vital signs check for BP, HR, and T	✘	✘	✘	✘	✘	✘	✘	✘	✘
Individual counseling	✘							✘	✘
Sociodemographic characteristics	✘								
Substance use history and service utilization	✘								
Severity of dependence scale (SDS)	✘								
Urine test for morphine, tramadol, methadone, buprenorphine, methamphetamine, benzodiazepines, and tetrahydrocannabinol	✘							✘	✘
Assessment of inclusion and exclusion criteria	✘								
Pregnancy test if female	✘								
Blood test for CBC, ALT, AST, PT, and PTT	✘								
HIV test	✘								
Informed consent form	✘								
Implementation of the opioid withdrawal management protocol	✘	✘	✘	✘	✘	✘	✘	✘	✘
Craving severity (NRS)	✘	✘	✘	✘	✘	✘	✘	✘	✘
Self-report of any drug use	✘	✘	✘	✘	✘	✘	✘	✘	✘
Subjective opioid withdrawal (SOWS)	✘	✘	✘	✘	✘	✘	✘	✘	✘
Clinical opioid withdrawal (COWS)	✘	✘	✘	✘	✘	✘	✘	✘	✘
Depression severity (BDI)	✘								
Quality of life (WHOQOL-BREF)	✘								
Treatment satisfaction in the intervention group (CSQ)								✘	
Treatment satisfaction in the comparison group (CSQ)									✘
Side effects in the intervention group		✘	✘	✘	✘	✘	✘	✘	
Side effects in the comparison group		✘	✘	✘	✘	✘	✘		✘
Naltrexone challenge test in the intervention group								✘	
Naltrexone challenge test in the comparison group									✘
Injection of XR-NTX in the intervention group								✘	
Injection of XR-NTX in the comparison group									✘

NA, naltrexone-accelerated; SB, sublingual buprenorphine; BP, blood pressure; HR, heart rate; T, temperature; ASI, addiction severity index; NRS, numerical rating scale; COWS, Clinical Opiate Withdrawal Scale; SOWS, Subjective Opiate Withdrawal Scale; BDI, Beck Depression Inventory; WHOQOL-BREF, World Health Organization quality of life; XR-NTX, extended-release injectable naltrexone; CSQ, client satisfaction questionnaire.

### 2.12 Protocol adherence and deviations

Protocol adherence is defined as attending daily visits, receiving the scheduled daily intervention, and completing all assessments. Any failure to comply with the procedures defined in the protocol—including absence from scheduled visits, violation of the prescribed medication regimen, violation of the inclusion or exclusion criteria identified after treatment initiation, any randomization error or mistake in treatment allocation, as well as any unintended initiation, discontinuation, or modification of treatment—is considered a deviation. All deviations are documented and categorized by the assessor. Deviations that have a substantial impact on the implementation of the intervention or the assessment of outcomes are classified as major deviations.

### 2.13 Analysis

All analyses will be conducted based on the intention-to-treat (ITT) principle. As a sensitivity analysis, a per-protocol analysis will be performed to assess the treatment effect in patients who adhered to the protocol. Missing data will be handled under the missing at random assumption, and a sensitivity analysis using multiple imputation will be performed to assess the robustness of the results.

Baseline characteristics of participants and baseline balance between the groups will be reported using descriptive statistics.

We will analyze primary outcomes using modified Poisson regression with robust standard errors. In this model, the treatment group and the variable used for stratified randomization (opioid-use category) will be entered as independent variables, and potential baseline confounders will be included as covariates when necessary.

Secondary outcomes, including craving severity, subjective and clinical withdrawal severity, safety, and opioid use, are measured repeatedly throughout the treatment period and will be analyzed using mixed-effects models. Continuous outcomes (craving severity, subjective and clinical withdrawal severity) will be analyzed using linear mixed-effects models with a Gaussian distribution and an identity link function, while binary outcomes (safety and opioid use) will be assessed using generalized linear mixed models with a binomial distribution and a logit link function. In all repeated-measures models, treatment group, time, the treatment group × time interaction, and the stratification variable used in randomization (opioid-use category) will be entered as fixed effects, and participant will be included as a random effect to account for within-participant correlation. When applicable, the baseline value of the outcome will also be included as a covariate. The covariance structure for repeated measures will be modeled as autoregressive of order 1 [AR(1)], with alternative covariance structures explored in sensitivity analyses if model convergence or data structure requires it.

Treatment satisfaction will be analyzed using linear regression. The treatment group and the variable used for stratification (opioid-use category) will be entered into the model.

Retention in treatment will be analyzed using survival analysis. In this analysis, all withdrawals from the study, regardless of the reason—such as revocation of informed consent, discontinuation of treatment, relapse, discontinuation of the injection, occurrence of adverse events or medical problems, loss to follow-up, return to opioid use during the second detoxification attempt, or three consecutive positive naltrexone challenges or urine tests—will be considered events. Patients receiving XR-NTR will be censored. Kaplan-Meier curves will be plotted for each treatment group, and the Cox proportional hazards model will be used to compare the groups. In this model, the treatment group and the stratification variable (opioid-use category) will be entered as fixed effects, and potential confounders will be included as covariates if necessary. The proportional hazards assumption will be tested, and if violated, the extended Cox model will be utilized.

### 2.14 Study monitoring and quality assurance

No independent Data Monitoring Committee (DMC) has been established for this trial, as the intervention is considered low-risk and the study is being conducted at a single center. Instead, all activities related to the monitoring and quality assurance of the study are conducted under the supervision of the principal investigator (PI), with the goal of maintaining the integrity of protocol implementation, ensuring participant safety, and enhancing the validity of the data. Given the comprehensive training provided to the research team during the pre-study phase, the monitoring process is designed based on a risk-based approach and is carried out through the following two main components:

#### 2.14.1 Internal audit

The implementation of the protocol is monitored by a trained internal auditor with prior research experience who has no involvement in the study execution, therapeutic decision-making, or data collection. After receiving specialized training on the study protocol, assessment tools, and the principles of Good Clinical Practice (GCP), the auditor is responsible for auditing the protocol implementation and reporting findings directly to the PI.

For this audit, at least 10% of each of the 8 treatment sessions are monitored. Sessions are randomly selected, followed by unannounced site visits by the auditor. The audit process involves direct observation of the treatment procedure and review of the CRFs, based on a dedicated session checklist. This level of monitoring is sufficient to identify significant protocol deviations, systematic errors in intervention implementation, and process-related issues in low-risk studies. Should systematic errors or major deviations be identified, the scope and intensity of monitoring are proportionally increased, and appropriate corrective and preventive measures are implemented accordingly.

#### 2.14.2 Data management and reporting

Data are manually entered into the Stata software by the assessor. To ensure confidentiality of information, each participant is assigned a unique code, and all data are recorded and analyzed only using this code. The PI randomly reviews approximately 5% of all entered data for accuracy. Any identified discrepancies are immediately corrected, and the correction process is documented in the study records. This review is conducted within the framework of data quality assurance and is distinct from the internal audit of protocol implementation. Access to the data file is restricted solely to the members of the core research team, and data protection is carried out according to the study’s data management protocol.

A professional and multidisciplinary team comprising a psychiatrist, two medical doctors, one clinical psychologist, one social worker, and one nurse will provide treatment services in accordance with standard procedures. A physician will be on site at all times in case treatment procedures cause distress to a patient. Individuals who experience an adverse event due to treatment interventions will receive treatment at the study site or, if needed, be referred to one of the TUMS-affiliated hospitals. A serious adverse event (SAE) is defined in accordance with the International Conference on Harmonisation E2A guideline (29). Within this trial, SAEs specifically include death, opioid overdose or severe opioid intoxication, precipitated withdrawal requiring hospitalization, severe hypersensitivity or anaphylactic reactions, clinically significant hepatotoxicity, serious suicidal ideation or suicide attempts, and any other medically significant event requiring hospitalization or urgent medical intervention. If an SAE occurs, we complete a dedicated report form and communicate it to the PI and the ethics committee within 24 hours. Necessary actions are then taken in accordance with research ethics requirements.

## 3 Discussion

This study is designed to compare the efficacy and safety of NA and SB withdrawal management. The aim is to determine which method facilitates patients’ transition to XR-NTX more effectively. This design allows researchers to accurately assess the primary and secondary outcomes associated with the withdrawal management process, providing valuable information for optimizing the treatment choice for patients with OUD.

From an international perspective, although the effectiveness of XR-NTX in improving treatment outcomes has been demonstrated in studies, the high rate of patient dropout before the injection remains one of the main barriers to the widespread use of this treatment approach. In other words, one of the most important challenges in providing XR-NTX treatment in outpatient settings is the completion of opioid withdrawal management and passage through the washout period. The development and evaluation of practical, safe, and feasible protocols to facilitate entering to XR-NTX treatment could play a crucial role in increasing patient access to antagonist-based treatments and expanding evidence-based treatment options globally.

Furthermore, this study has national importance. As discussed earlier, OAMT has become the dominant approach for treating OUD in Iran over the past few decades. Meanwhile, antagonist-based methods, including XR-NTX, despite their considerable clinical potential, have been used less frequently. The reasons for this are the limited availability of locally derived comparative evidence on the safety and efficacy of different withdrawal management methods, and low levels of clinicians’ knowledge and skills in evidence-based withdrawal approaches. The results of this study could contribute to generating credible local evidence and serve as a basis for clinical decision-making, therapist training, and the development of clinical guidelines adapted to the national healthcare system.

In this study, certain potential limitations must be considered. Among the most significant limitations are the lack of blinding and the presence of subjective outcomes, which may lead to participant/reporting bias, observer bias, and performance bias. To address these biases, the primary outcomes of the study are objective in nature and are not influenced by them. Additionally, the assessment of subjective outcomes, such as COWS, SOWS, NRS, and the CSQ questionnaire, is conducted by trained personnel in accordance with established protocols and standard procedures. Furthermore, the number and timing of visits in both groups have been predetermined and standardized as much as possible. However, despite these measures, complete elimination of biases is not feasible, and this should be taken into account when interpreting the results. Another limitation of the study is its single-center design, which may restrict demographic diversity and reduce the generalizability of the findings. Therefore, conducting future studies as multicenter trials with blinded designs could help reduce biases and enhance the generalizability of the results.

## References

[1] Zongyi Y Dongying C Yanyan S Xiaowu L . Global burden on drug use disorders from 1990 to 2021 and projections to 2046. Front Public Health. (2025) 13:1550518. doi: 10.3389/fpubh.2025.1550518 40791629 PMC12336172

[2] Fang M Zhang Q Peng J Yao W Feng W Wan X . Global, regional, and national burden of opioid use disorder from 1990 to 2021: a statistical analysis of incidence, mortality, and disability-adjusted life years. BMC Public Health. (2025) 25:1. doi: 10.1186/s12889-025-23283-1 40442654 PMC12121005

[3] World Health Organization . WHO Updates Guidelines on Opioid Dependence Treatment and Overdose Prevention. Geneva: WHO (2025). Available online at: https://www.who.int/news/item/09-02-2025-who-updates-guidelines-on-opioid-dependence-treatment-and-overdose-prevention?utm_source=chatgpt.com (Accessed September 24, 2026).

[4] Bisaga A Mannelli P Sullivan MA Vosburg SK Compton P Woody GE . Antagonists in the medical management of opioid use disorders: historical and existing treatment strategies. Am J Addict. (2018) 27:3. doi: 10.1111/ajad.12711 29596725 PMC5900907

[5] Minozzi S Amato L Vecchi S Davoli M Kirchmayer U Verster A . Oral naltrexone maintenance treatment for opioid dependence. Cochrane Database Syst Rev. (2011) 4:Cd001333. doi: 10.1002/14651858.CD001333.pub4 21491383 PMC7045778

[6] U.S. Food and Drug Administration . Vivitrol (Naltrexone for Extended-Release Injectable Suspension) Approval Letter (2010). Available online at: https://www.accessdata.fda.gov/drugsatfda_docs/appletter/2010/021897s015ltr.pdf (Accessed September 24, 2026).

[7] Nunes EV Bisaga A Krupitsky E Nangia N Silverman BL Akerman SC . Opioid use and dropout from extended‐release naltrexone in a controlled trial: implications for mechanism. Addiction. (2020) 115:2. doi: 10.1111/add.14735 31313402 PMC6980175

[8] Krupitsky E Nunes EV Ling W Illeperuma A Gastfriend DR Silverman BL . Injectable extended-release naltrexone for opioid dependence: a double-blind, placebo-controlled, multicentre randomised trial. Lancet. (2011) 377:1506–13. doi: 10.1016/S0140-6736(11)60358-9 21529928

[9] Lee JD Nunes EV Novo P Bachrach K Bailey GL Bhatt S . Comparative effectiveness of extended-release naltrexone versus buprenorphine-naloxone for opioid relapse prevention (X: BOT): a multicentre, open-label, randomised controlled trial. Lancet. (2018) 391:309–18. doi: 10.1016/S0140-6736(17)32812-X 29150198 PMC5806119

[10] Srivastava AB Mariani JJ Levin FR . New directions in the treatment of opioid withdrawal. Lancet. (2020) 395:1938–48. doi: 10.1016/S0140-6736(20)30852-7 32563380 PMC7385662

[11] Mordal J Juya F Holtan L Vederhus J-K Opheim A Brenna IH . High induction rate onto extended-release naltrexone for people with opioid use disorder: experiences from a Norwegian naturalistic study. Addict Sci Clin Pract. (2025) 20:1. doi: 10.1186/s13722-025-00576-9 40524244 PMC12168299

[12] Dunn KE Sigmon SC Strain EC Heil SH Higgins ST . The association between outpatient buprenorphine detoxification duration and clinical treatment outcomes: a review. Drug Alcohol Depend. (2011) 119:1–9. doi: 10.1016/j.drugalcdep.2011.05.033 21741781 PMC3205338

[13] Day E Strang J . Outpatient versus inpatient opioid detoxification: a randomized controlled trial. J Subst Abuse Treat. (2011) 40:1. doi: 10.1016/j.jsat.2010.08.007 21036514

[14] Sullivan M Bisaga A Pavlicova M Choi CJ Mishlen K Carpenter KM . Long-acting injectable naltrexone induction: a randomized trial of outpatient opioid detoxification with naltrexone versus buprenorphine. Am J Psychiatry. (2017) 174:5. doi: 10.1176/appi.ajp.2016.16050548 28068780 PMC5411308

[15] Comer SD Mannelli P Alam D Douaihy A Nangia N Akerman SC . Transition of patients with opioid use disorder from buprenorphine to extended‐release naltrexone: a randomized clinical trial assessing two transition regimens. Am J Addict. (2020) 29:4. doi: 10.1111/ajad.13024 32246728 PMC7383475

[16] Bisaga A Mannelli P Yu M Nangia N Graham CE Tompkins DA . Outpatient transition to extended-release injectable naltrexone for patients with opioid use disorder: A phase 3 randomized trial. Drug Alcohol Depend. (2018) 187:171–8. doi: 10.1016/j.drugalcdep.2018.02.023 29674251

[17] Levin FR Mariani JJ Pavlicova M Choi CJ Basaraba C Mahony AL . Lorcaserin treatment for extended-release naltrexone induction and retention for opioid use disorder individuals: A pilot, placebo-controlled randomized trial. Drug Alcohol Depend. (2021) 219:108482. doi: 10.1016/j.drugalcdep.2020.108482 33418204 PMC7856237

[18] Habibi M Alahdadi S Ramezani Piyani M . Psychometric properties of severity of dependence scale (SDS) in people with drug and alcohol dependency. Q J Res Addict. (2018) 12:46. In Persian.

[19] Wewers ME Lowe NK . A critical review of visual analogue scales in the measurement of clinical phenomena. Res Nurs Health. (1990) 13:4. doi: 10.1002/nur.4770130405 2197679

[20] Wesson DR Ling W . The clinical opiate withdrawal scale (COWS). J Psychoactive Drugs. (2003) 35:2. doi: 10.1080/02791072.2003.10400007 12924748

[21] Ziaaddini H Nasirian M Nakhaee N . Comparison of the efficacy of Buprenorphine and Clonidine in detoxification of opioid-dependents. Addict Health. (2012) 4:79–86. PMC3905537 24494140

[22] Handelsman L Cochrane KJ Aronson MJ Ness R Rubinstein KJ Kanof PD . Two new rating scales for opiate withdrawal. Am J Drug Alcohol Abuse. (1987) 13:3. doi: 10.3109/00952998709001515 3687892

[23] The WHOQOL Group . Development of the World Health Organization WHOQOL-BREF quality of life assessment. Psychol Med. (1998) 28:3. doi: 10.1017/s0033291798006667 9626712

[24] Nejat SN Montazeri A Holakouei Naeini K Mohammad K Majdzadeh SR . The World Health Organization quality of Life (WHOQOL-BREF) questionnaire: Translation and validation study of the Iranian version. sjsph. (2006) 4:1–12.

[25] Dozois DJA . Beck depression inventory-II. In: Weiner IB Craighead WE , editors. The Corsini Encyclopedia of Psychology, Volume 1. John Wiley & Sons, Inc, Hoboken, New Jersey (2010). p. 210–11.

[26] Ghassemzadeh H Mojtabai R Karamghadiri N Ebrahimkhani N . Psychometric properties of a Persian‐language version of the Beck Depression Inventory‐Second edition: BDI‐II‐PERSIAN. Depress Anxiety. (2005) 21:4. doi: 10.1002/da.20070 16075452

[27] Larsen DL Attkisson CC Hargreaves WA Nguyen TD . Assessment of client/patient satisfaction: development of a general scale. Eval Program Plann. (1979) 2:3. doi: 10.1016/0149-7189(79)90094-6 10245370

[28] Sharifi V Tehranidoost M Yunesian M Amini H Mohammadi M Jalali Roudsari M . Effectiveness of a low-intensity home-based aftercare for patients with severe mental disorders: a 12-month randomized controlled study. Community Ment Health J. (2012) 48:6. doi: 10.1007/s10597-012-9516-z 22772746

[29] Guideline IHT . Clinical safety data management: definitions and standards for expedited reporting E2A. In: International Conference on Harmonisation of Technical Requirements for Registration of Pharmaceuticals for Human Use Geneva, Switzerland: International Conference on Harmonisation (ICH). (1994). Available online at: http://fercsl.lk/wp/wp-content/uploads/2019/04/Clinical-Safety-Data-Management-Definitions-Standards-for-Expedited-Reporting-ICH-Harmonised-Tripartite-Guideline-1994.pdf (Accessed September 24, 2026).

